# Cyclic GMP is involved in auxin signalling during *Arabidopsis* root growth and development

**DOI:** 10.1093/jxb/eru019

**Published:** 2014-03-03

**Authors:** Wenbin Nan, Xiaomin Wang, Lei Yang, Yanfeng Hu, Yuantao Wei, Xiaolei Liang, Lina Mao, Yurong Bi

**Affiliations:** Key Laboratory of Cell Activities and Stress Adaptations, Ministry of Education, School of Life Sciences, Lanzhou University, Lanzhou, Gansu 730000, People’s Republic of China

**Keywords:** *Arabidopsis thaliana*, auxin signalling, cGMP, guanylate cyclase, PKG, TIR1-Aux/IAA interaction.

## Abstract

The second messenger cyclic guanosine 3′,5′-monophosphate (cGMP) plays an important role in plant development and responses to stress. Recent studies indicated that cGMP is a secondary signal generated in response to auxin stimulation. cGMP also mediates auxin-induced adventitious root formation in mung bean and gravitropic bending in soybean. Nonetheless, the mechanism of the participation of cGMP in auxin signalling to affect these growth and developmental processes is largely unknown. In this report we provide evidence that indole-3-acetic acid (IAA) induces cGMP accumulation in *Arabidopsis* roots through modulation of the guanylate cyclase activity. Application of 8-bromo-cGMP (a cell-permeable cGMP derivative) increases auxin-dependent lateral root formation, root hair development, primary root growth, and gene expression. In contrast, inhibitors of endogenous cGMP synthesis block these processes induced by auxin. Data also showed that 8-bromo-cGMP enhances auxin-induced degradation of Aux/IAA protein modulated by the SCF^TIR1^ ubiquitin-proteasome pathway. Furthermore, it was found that 8-bromo-cGMP is unable to directly influence the auxin-dependent TIR1-Aux/IAA interaction as evidenced by pull-down and yeast two-hybrid assays. In addition, we provide evidence for cGMP-mediated modulation of auxin signalling through cGMP-dependent protein kinase (PKG). Our results suggest that cGMP acts as a mediator to participate in auxin signalling and may govern this process by PKG activity via its inﬂuence on auxin-regulated gene expression and auxin/IAA degradation.

## Summary

This work demonstrated the crosstalk between cGMP signalling and auxin signalling and evidenced that cGMP affects the auxin signalling through the PKG activity in *Arabidopsis* root development.

## Introduction

The plant hormone auxin plays a central role in plant responses to physiological and environmental changes. It regulates cell division and differentiation, embryogenesis, organogenesis, phototropic and gravitropic responses, and root and shoot architecture formation (Woodward and Bartel, 2005; Vanneste and Friml, 2009). Optimal post-embryonic root growth requires tight control of indole-3-acetic acid (IAA) activity, which can be regulated by diverse mechanisms including IAA biosynthesis, its transport, and signal transduction (Hayashi, 2012).

It has been demonstrated that the auxin signal transduction system operates via the SCF^TIR1/AFB^ proteasome machinery (TIR1/AFB is TRANSPORT INHIBITOR RESPONSE1/AUXIN RECEPTOR-BOX), which plays a key role in the regulatory process of transcription and leads to auxin-related developmental responses (Chapman and Estelle, 2009). In this process, auxin promotes the degradation of auxin/indole-3-acetic acid (Aux/IAA) transcriptional repressors through the proteasome pathway by enhancing the ubiquitination of Aux/IAA proteins (Gray *et al.*, 2001). Aux/IAAs are recognized as substrates and the F-box protein TIR1 is a component of the SCF^TIR1/AFB^-type E3 ubiquitin ligase complex (Kepinski, 2007). Furthermore, TIR1 and ﬁve TIR1 homologue proteins (AFB1–AFB5) redundantly function as nuclear auxin receptors in *Arabidopsis* (Dharmasiri *et al.*, 2005*a*; Kepinski and Leyser, 2005; Parry *et al.*, 2009; Greenham *et al.*, 2011). Recent studies demonstrated that auxin acts as a ‘molecular glue’ in binding to TIR1 and stabilizing the interaction between TIR1 and Aux/IAA proteins (Santner and Estelle, 2009; Greenham *et al.*, 2011). This interaction results in Aux/IAA ubiquitination and subsequent degradation by the 26S proteasome and therefore releases the AUXIN RESPONSE FACTOR (ARF) proteins to regulate the expression of target genes (Tan *et al.*, 2007). Auxin rapidly alters the expression of hundreds of genes within minutes and early auxin-inducible genes are classiﬁed into three major families: *SAUR* (*Small auxin up RNA*), *GH3* (*Gretchen Hagen3*), and *AUX/IAA* genes (Goda *et al.*, 2008; Chapman and Estelle, 2009). The *Arabidopsis* Aux/IAA family comprises 29 members, which encode short-lived nuclear proteins that function as unstable repressors regulating auxin-inducible gene expression (Worley *et al.*, 2000; Remington *et al.*, 2004). When auxin levels are low, Aux/IAAs are relatively stable and able to exert repression on target genes. As auxin levels rise, TIR1-mediated proteolysis of Aux/IAAs relieves the repression, and target genes are expressed. The SCF regulatory proteins AXR1, ECR1, and RCE1 are involved in the RUB/NEDD8 conjugation of CUL1. Mutations in these components confer auxin-resistant phenotypes and result in defects in auxin-related developmental processes (del Pozo *et al.*, 2002; Dharmasiri *et al.*, 2003). Auxin signalling components have been conserved throughout the evolution of land plants and have proliferated and specialized to control speciﬁc developmental processes (Chapman and Estelle, 2009).

It has been shown that the second messenger cyclic guanosine 3′,5′-monophosphate (cGMP) is an important signalling component with multiple biological functions in plants (Newton and Smith, 2004), similar to the situation in animals. cGMP has been detected in several plant species, including barley, mung bean, tobacco, soybean, and *Arabidopsis* (Penson *et al.*, 1996; Donaldson *et al.*, 2004; Hu *et al.*, 2005; Bai *et al.*, 2012). Cellular cGMP level has been measured using the ﬂuorescent reporter FlincG as an endogenous cGMP sensor (Isner and Maathuis, 2011; Isner *et al.*, 2012). The cGMP signalling pathway and its feedback regulation operate by a combination of cGMP synthesis and degradation through guanylate cyclase (GC) and 3′,5′-cyclic-cGMP phosphodiesterase, respectively (Meier and Gehring, 2006). In *Arabidopsis*, it has been reported that six proteins, GC (AtGC1), wall-associated kinase-like protein (AtWAKL10), brassinosteroid receptor (AtBRI1), Pep1 receptor (AtPepR1), phytosulfokine receptor (AtPSKR), and nitric oxide-binding GC (AtNOGC1), show the GC activity, thus generating cGMP from GTP *in vitro* and *in vivo* (Wong and Gehring, 2013). cGMP functions by regulating cGMP-dependent protein kinases (PKGs), cGMP-regulated phosphodiesterases, and cyclic nucleotide-gated ion channels (Potter *et al.*, 2006). In plants, there is some biochemical evidence supporting the existence of plant cGMP-responsive kinases (Newton and Smith, 2004), and the *Arabidopsis* genome contains sequences that encode gene products with both a cyclic nucleotide-binding domain and a protein kinase (Meier and Gehring, 2006). However, speciﬁc cGMP targets in plants are largely unknown and in particular there is little molecular evidence available of bonaﬁde cGMP-dependent kinases.

In plants, cGMP is involved in stress responses, seed germination (Teng *et al.*, 2010), α-amylase production (Penson *et al.*, 1996; Wu *et al.*, 2013), stomatal movement (Dubovskaya *et al.*, 2011; Joudoi *et al.*, 2013), reorientation of pollen tube and cell polarity (Prado *et al.*, 2004; Salmi *et al.*, 2007), and anthocyanin and ﬂavonoid biosynthesis (Bowler *et al.*, 1994; Suita *et al.*, 2009). Moreover, cGMP plays a role in plant responses to various phytohormones, including gibberellic acid, auxin, and abscisic acid (Penson *et al.*, 1996; Pagnussat *et al.*, 2003; Dubovskaya *et al.*, 2011). In some of these responses, cGMP is the downstream molecule of phytohormone action and mediates phytohormone signalling, suggesting possible crosstalk between phytohormones and cGMP. In particular, cGMP is an important molecule in auxin-regulated signalling in determining root morphology during growth and development (Bai *et al.*, 2012). cGMP accumulation was reported in response to auxin treatment during adventitious root formation and asymmetric cGMP accumulation in root tips during the gravitropic response (Pagnussat *et al.*, 2003; Hu *et al.*, 2005; Bai *et al.*, 2012). Auxin-induced adventitious roots and root gravitropic responses were blocked by GC inhibitors 6-anilino-5,8-quinolinedione (LY83583) or 1H-[1,2,4]-oxadiazole-[4,3-a]-quinoxalin-1-one (ODQ), suggesting a key role for endogenous cGMP in these processes (Pagnussat *et al.*, 2003; Hu *et al.*, 2005; Bai *et al.*, 2012). However, the molecular mechanism of cGMP and auxin interaction in the root development of plant is still poorly understood.

In the study we used auxin-related mutants and transgenic plants to analyse how cGMP is involved in the auxin signalling and thus affects root growth and development in *Arabidopsis*. Our results demonstrated that cGMP modulated auxin-dependent gene expression and Aux/IAA protein degradation through the stimulation of PKG activity. The results presented here provide evidence for a link between the auxin signalling pathway and the cGMP signalling pathway.

## Materials and methods

### Plant materials, growth conditions, and chemicals

The *Arabidopsis* mutants *tir1-1* (Ruegger *et al.*, 1998), *axr1-3*, and *axr1-12* (Lincoln *et al.*, 1990) and the transgenic lines *DR5*::*GUS* (Ulmasov *et al.*, 1997), *HS*::*AXR3NT-GUS*, *HS*::*axr3-1NT-GUS*, and *HS*::*GUS*, myc-*TIR1* (Gray *et al.*, 2001) have been previously described, and all of them are in the Col-0 background. Seeds were sterilized with 1.5% NaClO for 15min, washed with sterile water three times, placed in 4 °C for 3 days and then planted on the half-strength Murashige and Skoog (1/2MS) medium (pH 5.8) containing 1% sucrose and 0.8% agar in the growth room at 23 °C under 100–120 μmol photons·m^−2^·s^−1^ with a 16h/8h light/dark photoperiod.

In the study, LY83583 and ODQ were used as the GC inhibitors and 8-bromoguanosine 3′,5′-cyclic guanosine monophosphate (8-Br-cGMP) was used as a cell-permeable cGMP derivative; Rp-8-Br-cGMP, MG132, and 1-naphthylphthalamic acid (NPA) were used as the PKG, proteasome, and auxin transport inhibitors, respectively. The above chemicals were purchased from Sigma-Aldrich (St Louis, MO, USA) except NPA (Chem Service, West Chester, PA, USA). They were contained in the medium for different treatments.

### Phenotypic analysis and statistics

The length of the primary roots and lateral roots (LRs) was measured with NIH Image software (Image J, version 1.43). Emerged LRs and β-glucuronidase (GUS)-staining sites were counted using an anatomical lens. Root hairs were photographed with a Leica stereo microscope and the density was counted in a 2.5mm region from the primary root tip.

### GUS staining and quantitative GUS activity assays

GUS staining was carried out according to the methods described by Pelagio-Flores *et al.* (2011) with some modiﬁcations. Brieﬂy, seedlings were ﬁxed in 90% acetone at −20 °C for 1h, washed twice in 50mM sodium phosphate buffer (pH 7.0) and then incubated in GUS-staining buffer containing 1mM 5-bromo-4-chloro-3-indolyl-β-D-glucuronic acid (X-Gluc), 100mM sodium phosphate (pH 7.5), 0.5mM K_3_[Fe(CN)_6_], 0.5mM K_4_[Fe(CN)_6_], 10mM EDTA, and 0.1% Triton X-100. The seedlings were incubated at 37 °C for 6–18h and then cleared using HCG solution (chloroacetaldehyde/water/glycerol = 8:3:1) for 12h. Individual representative seedlings were photographed using a Leica Microsystems DM5000B microscope.

Quantitative GUS activity assay was performed as described by Hu *et al.* (2012). Root samples were homogenized in GUS extraction buffer (50mM potassium phosphate buffer, pH 7.0, 10mM EDTA, 0.1% Triton X-100, and 0.1% SDS). The extract was centrifuged at 12000 *g* for 15min at 4 °C. The ﬂuorogenic reaction was carried out in a reaction mixture containing 2mM 4-methylumbelliferyl-d-glucuronide (MUG; Sigma-Aldrich) as a substrate and 80 μg of total protein in ﬁnal volume of 0.5ml at 37 °C for 30min, and then the reaction was terminated with the addition of 0.2M Na_2_CO_3_. Fluorescence was measured with excitation at 365nm and emission at 455nm on a Thermo Scientiﬁc NanoDrop 2000c spectroﬂuorimeter. Enzyme activity was calibrated by standard curve of 4-methylumbelliferone (4-MU; Sigma-Aldrich). Protein content was normalized according to the method of Bradford (1976).

### Quantitative real-time PCR analysis

Total RNA was extracted with Trizol (TaKaRa) from roots, and then was treated with RNase-free DNase (Promega, Madison, WI, USA). First-strand cDNA was synthesized with the PrimeScript II 1st Strand cDNA Synthesis Kit (TaKaRa, Mountain View, CA, USA). Quantitative real-time PCR was performed using the SYBR PrimeScript RT-PCR Kit (Perfect Real Time; TaKaRa). PCR was performed using a CFX 96 Real-Time system (Bio-Rad, Hercules, CA, USA) with the following standard cycling conditions: 95 °C for 10 s, followed by 40 cycles of 95 °C for 5 s, and 60 °C for 30 s. The cycle threshold 2^(−ΔΔC(T))^-based method was used for relative quantitation of gene expression. The speciﬁc primers for each gene are listed in Table S1. Expression levels of genes were normalized to *ACTIN2* levels.

### cGMP content and GC activity assay

For cGMP content assay, 200mg roots were ground in liquid N_2_. Then 1.5ml of ice-cold 6% (v/v) trichloroacetic acid was added, and the homogenate was centrifuged at 1000 *g* for 15min at 4 °C. The supernatant was extracted four times in five volumes of water-saturated diethyl ether. The aqueous extract was dried under a stream of N_2_ at 60 °C and stored at −70 °C. The cGMP content was measured according to the manufacturer’s protocol of cGMP enzyme immunoassay kit (Sigma-Aldrich). The standard curve is presented in Tables S2 and S3 and Fig. S1.

For the GC activity assay, roots were homogenized in a medium containing 175mM Tris/HCl (pH 7.9), 20mM theophylline, and a protease inhibitor cocktail for plant cell and tissue extracts (Sigma-Aldrich). The homogenate was centrifuged at 1300 *g* for 5min at 4 °C. GC activity was measured by estimating the rate of cGMP formation from Mn^2+^-GTP in a reaction mixture containing 175mM Tris/HCl (pH 7.9), 20mM theophylline, 20mM MnCl_2_, 1mM GTP, and 60 μg of total protein in ﬁnal volume of 0.25ml (Dubovskaya *et al.*, 2011). The reaction mixture was incubated at 25 °C for 10min. Then 0.25ml of 0.2M Na_2_CO_3_ was added and the solution was mixed, frozen at −70 °C, thawed, mixed again, and centrifuged at 6000 *g* for 10min. cGMP content in the supernatant was measured using cGMP enzyme immunoassay kit.

### Proteasome assay

The ATP-dependent 20S core unit activity of the 26S proteasome in 7-day-old wild-type (WT) seedlings was measured by peptide hydrolysis activity using succinyl-Leu-Leu-Val-Tyr-4-methyl-coumaryl-7-amide (Sigma-Aldrich) as the substrate with or without ATP and Mg^2+^ (Fujinami *et al.*, 1994). Brieﬂy, roots were homogenized with 50mM Tris/HCl buffer (pH 8.0) containing 20mM 2-mercaptoethanol. The extract was centrifuged at 12000 *g* for 15min at 4 °C. The supernatant was added to the reaction solution (50mM Tris/HCl buffer, 20mM 2-mercaptoethanol, and 10mM substrate with or without 4mM ATP and 10mM MgCl_2_) and then the mixture was incubated for 50min at 37 °C and stopped with 0.2M Na_2_CO_3_. The ﬂuorescence from the hydrolysed substrate was measured using a ﬂuorometer (excitation 380nm, emission 440nm).

### Yeast two-hybrid assay

The vectors and strains used for the yeast two-hybrid assay were provided in the Matchmaker *GAL4* Two-Hybrid System 3 (Clontech, Mountain View, CA, USA). The yeast two-hybrid assay was performed according to the *Yeast Protocol Handbook* (Clontech). The AD-IAA3, AD-IAA7, AD-IAA17, and BD-TIR1 plasmids were constructed by inserting PCR fragments of full-length cDNAs into the appropriate plasmids. The PCR fragments of IAA3, IAA7, IAA17, and TIR1 were amplified with specific primers containing an EcoRΙ or BamHI site. The resultant fragment was digested with EcoRΙ and BamHI, and cloned into pGADT7 and pGBKT7 to generate plasmid AD-IAA3, AD-IAA7, AD-IAA17, and BD-TIR1. The plasmids were co-transformed into yeast strain AH109. All primers used for yeast two-hybrid assays are listed in Table S1.

### Pull-down assay

Pull-down assays with bacterially produced glutathione-S-transferase (GST)-IAA7/AXR2 were performed as described previously (Gray *et al.*, 2001). Brieﬂy, 100 μl of TIR1-Myc protein extracted from 7-day-old seedlings of the transgenic line *tir1-1*[TIR1-Myc] was incubated for 2.5h at 4 °C with >50 μg of glutathione-agarose beads (Sigma-Aldrich) on which GST-IAA7/AXR2 protein was immobilized in 500 μl of reaction buffer (20mM Tris pH 8.0, 200mM NaCl, and 5mM dithiothreitol) in the presence of the indicated compounds. After incubation at 4 °C the agarose beads were collected by brief centrifugation, washed three times, and suspended in SDS/PAGE sample buffer. The bound proteins were separated by SDS/PAGE and interacting TIR1-Myc was detected by immunoblotting with anti-Myc antibodies.

### Statistical analysis

Each experiment was repeated at least three times. Values are expressed as mean ± SE. For all experiments the overall data were statistically analysed using SPSS version 17.0. All comparisons were performed using one-way analysis of variance with Duncan’s test or Tukey’s test for independent samples. In all cases the conﬁdence coefﬁcient was set at *P* < 0.05.

## Results

### Auxin increases the levels of endogenous cGMP in *Arabidopsis* roots

**Fig. 1.**
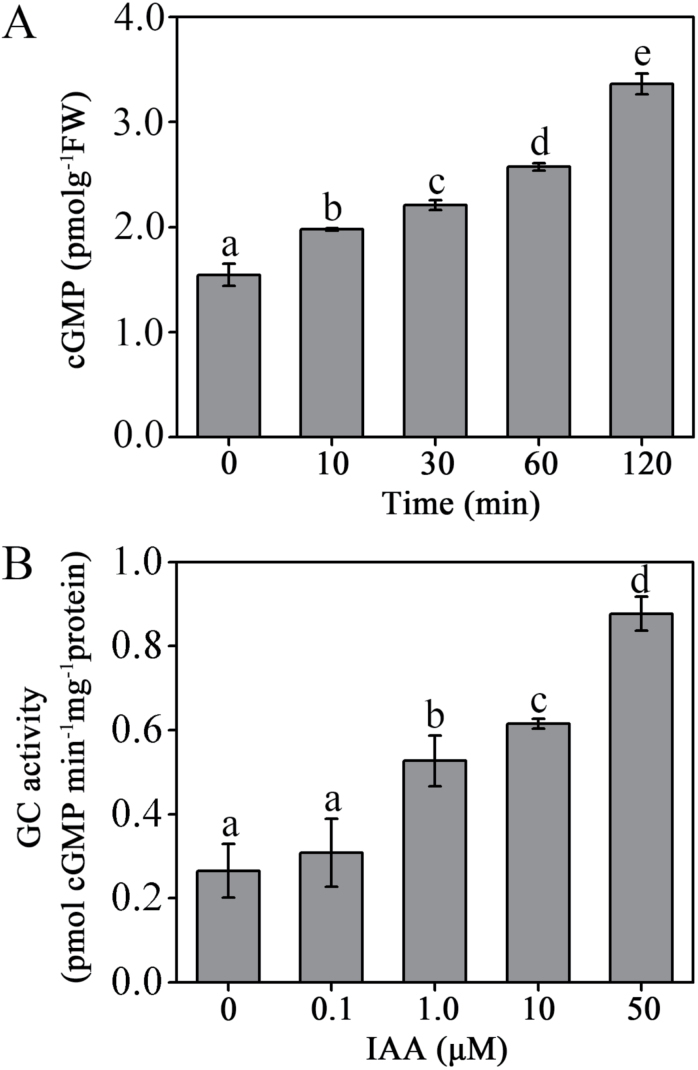
IAA increases endogenous cGMP content (A) and GC activity (B) in *Arabidopsis* roots. Seven-day-old WT seedlings were incubated in 5 μM IAA for 10–120min (A) or 0.1–50 μM IAA for 1h (B). Mean values and SE were calculated from three independent experiments.

It has been indicated that cGMP is a secondary signal that acts in response to auxin stimulation and mediates auxin-induced adventitious root formation in mung bean and gravitropic bending in soybean. To further explore the mechanism of cGMP involvement in auxin signalling responses, we measured cGMP production in *Arabidopsis* roots. Results showed that cGMP levels were markedly induced in 5 μM auxin treatments for 10–120min, and that they increased to 217.7% of the control after 5 μM auxin treatment for 120min (Fig. 1A, Fig. S2). To investigate how auxin increases the endogenous cGMP level, the GC activity was examined. As shown in Fig. 1B, IAA stimulated GC activity, and it increased from 116.2 to 330.7% of the control under 0.1–50 μM IAA treatments for 1h. These results suggested that IAA induces cGMP levels by stimulating the GC activity.

### Auxin-induced development of the root system is cGMP-dependent in *Arabidopsis*

**Fig. 2.**
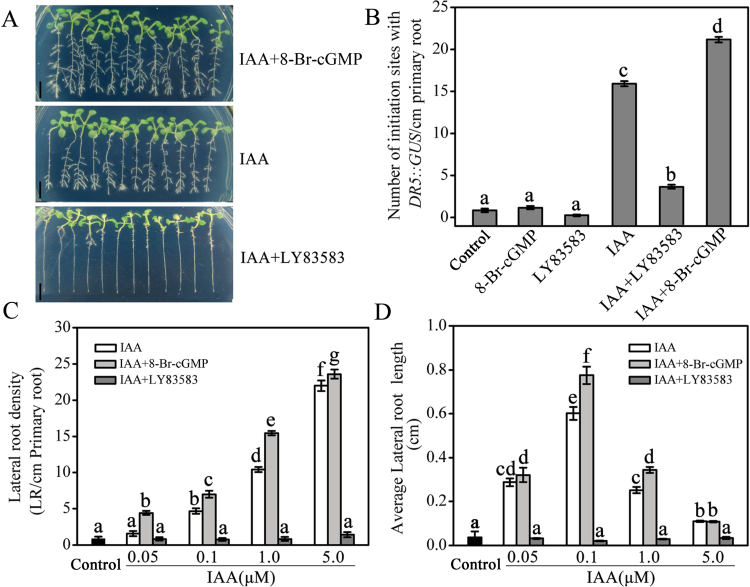
Effects of cGMP on auxin-induced LR formation. (A) Photographs of LR formation in WT seedlings after 5 days and (B) the number of *DR5*::*GUS*-staining sites in the transgenic line *DR5::GUS* for 2 days under 1 μM IAA, 100 μM 8-Br-cGMP, and 20 μM LY83583 treatments as indicated. (C) Changes in LR density and (D) average LR length under 0.05–5.0 μM IAA, IAA plus 100 μM 8-Br-cGMP, and IAA plus 20 μM LY83583 treatments after 5 days in WT seedlings. WT and *DR5*::*GUS* seedlings were grown on medium containing 0.5 μM NPA for 5 days to repress LR initiation, and then they were treated with IAA, 8-Br-cGMP, or LY83583. Mean values and SE were calculated from three independent experiments (*n* = 12). Within each set of experiments, bars with different letters were signiﬁcantly different at the 0.05 level. Scale bar in A, 250mm. This figure is available in colour at JXB online.

We next used 8-Br-cGMP, a cell-permeable cGMP derivative, and LY83583, a GC inhibitor, to investigate the roles of cGMP in auxin-induced root-system development in *Arabidopsis* roots, including the LR formation, root hair development, and the inhibition of primary root elongation. As shown in Fig. 2A and C, 0.1–5.0 μM IAA treatment for 5 days markedly increased LR density to 1.4–19 times of the control, respectively. The effects of IAA were obviously strengthened by co-treatment with 100 μM 8-Br-cGMP. In contrast, the LR density induced by auxin was completely suppressed by 20 μM LY83583 (Fig. 2A, C). Interestingly, we also observed that 8-Br-cGMP co-treatment with IAA increased the length of LRs and LY83583 co-treatment with IAA markedly decreased it in comparison with IAA treatment alone (Fig. 2A, D). However, there was no LR formation in seedlings treated alone with 8-Br-cGMP or LY83583 after another 5 days of treatment (data not shown).

To determine whether cGMP promotion of auxin-induced LR formation occurs prior to the emergence of LRs, we employed the *DR5*::*GUS* auxin-responsive reporter (Ulmasov *et al.*, 1997) as a molecular probe. *DR5*::*GUS* is expressed in the dividing pericycle cells during LR initiation and indicates the initial cells where LR initiation occurs (Benkova *et al.*, 2003). Our results showed that the number of IAA-induced LR primordium sites in *DR5*::*GUS* was signiﬁcantly increased by 8-Br-cGMP and decreased by LY83583 (Fig. 2B). However, the application of 8-Br-cGMP or LY83583 alone did not produce any different effects for the number of *DR5*::*GUS* sites compared to controls. Taken together, these results indicate that cGMP is required for auxin-stimulated LR formation as well as LR initiation.

**Fig. 3.**
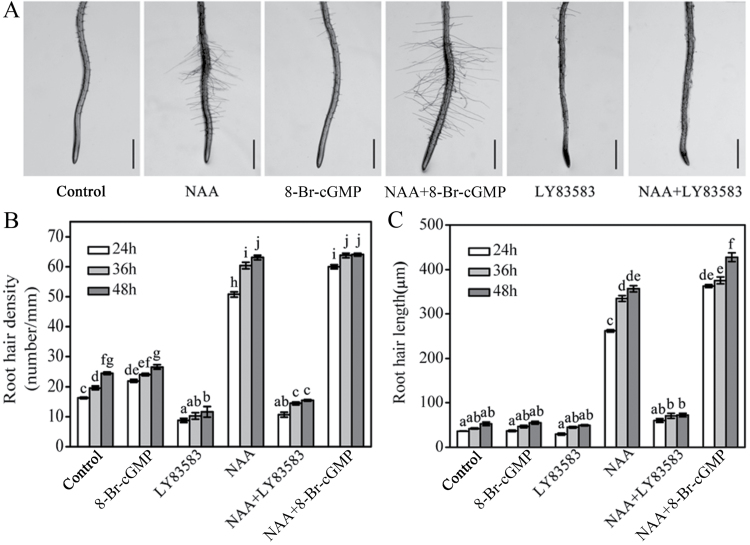
Effects of cGMP on the auxin-induced root hair development in WT seedlings. (A) Photographs of root hairs formed under various treatments for 36h in the primary root tip. Scale bar, 500 μm. (B) The changes of root hair density and (C) root hair length under various treatments for 24–48h. The 5-day-old seedlings were transferred onto vertical plates containing 0.1 μM NAA, 100 μM 8-Br-cGMP, or 20 μM LY83583 for the various treatments. Mean values and SE were calculated from three independent experiments (*n* = 20). Within each set of experiments, bars with different letters were signiﬁcantly different at the 0.05 level. This figure is available in colour at JXB online.

The root hair density and the root hair length also dramatically increased under 0.1 μM 1-naphthaleneacetic acid (NAA) treatments for 24–48h (Fig. 3). Application of 8-Br-cGMP or LY83583 in combination with NAA treatment dramatically enhanced or inhibited the auxin-induced development of root hairs, including auxin-stimulated initiation and elongation of root hairs compared with the NAA treatment alone (Fig. 3). 8-Br-cGMP treatment alone exhibited a weak increase in root hair density, but not in root hair length (Fig. 3A-C). These results indicate that cGMP is also involved in auxin-regulated root hair development.

**Fig. 4.**
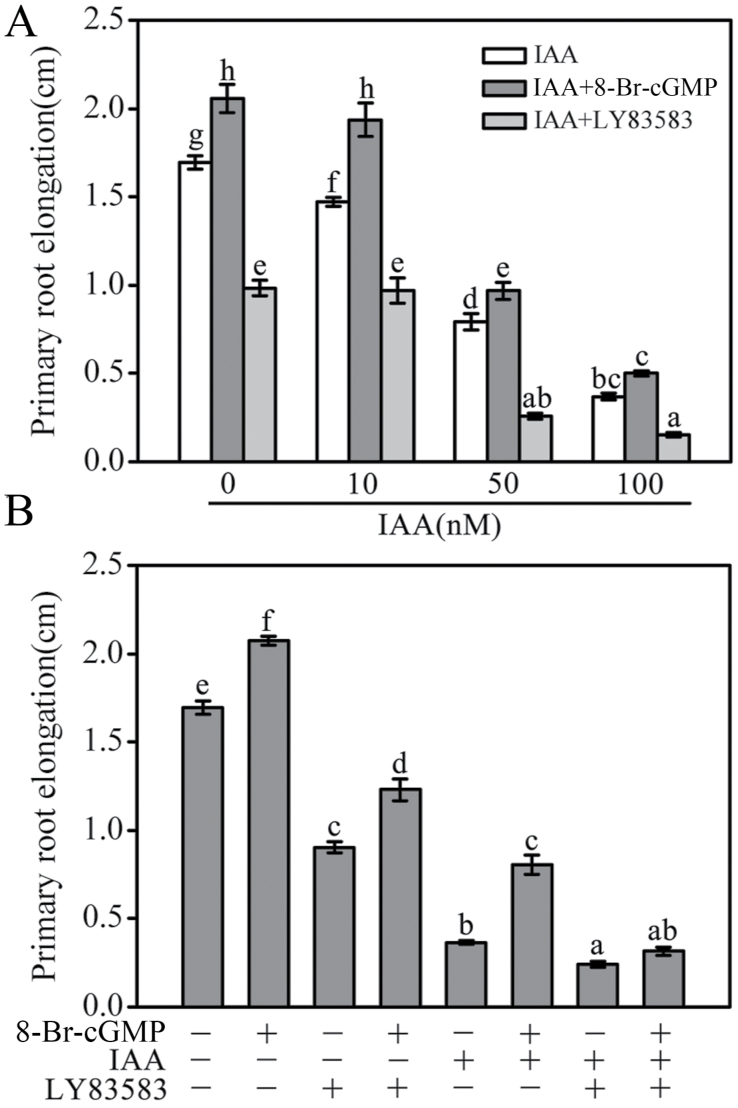
Effects of cGMP on auxin-inhibited primary root elongation in WT seedlings. The 5-day-old seedlings were transferred onto the vertical plates containing (A) 0–100nM or (B) 50nM IAA, and 100 μM 8-Br-cGMP and/or 2.5 μM LY83583 as shown, for various treatments for 3 days. Mean values and SE were calculated from three independent experiments (*n* = 20). Within each set of experiments, bars with different letters were signiﬁcantly different at the 0.05 level.

As shown in Fig. 4, 100 μM 8-Br-cGMP treatment alone for 3 days slightly increased the *Arabidopsis* primary root elongation (by 122.4%) compared with the control, whereas 2.5 μM LY83583 signiﬁcantly suppressed it (by 53.3%). 8-Br-cGMP treatment partially reversed the effect of LY83583, suggesting that cGMP is essential for the normal growth of *Arabidopsis* primary roots (Fig. 4B). The present data also showed that 10–100nM IAA treatment markedly reduced primary root elongation (86.9–21.8% of the control) and that application of 8-Br-cGMP or LY83583 rescued or aggravated the action of IAA (Fig. 4A, B). Furthermore, responses induced by LY83583 plus IAA were partially reversed by co-treatment with 8-Br-cGMP (Fig. 4B). Together, the pharmacological data revealed that cGMP plays an important role in auxin-induced root-system development in *Arabidopsis* roots.

### Effects of cGMP on the auxin-inhibited primary root elongation in auxin-related *Arabidopsis* mutants

**Fig. 5.**
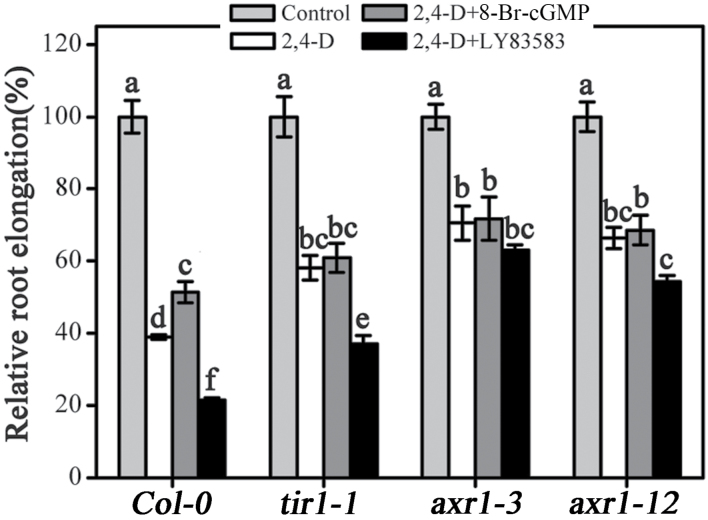
Effects of cGMP on the auxin-inhibited primary root elongation in *Arabidopsis tir1-1*, *axr1-3*, and *axr1-12* mutants and WT. The 5-day-old seedlings were transferred onto vertical plates containing 30nM 2,4-D, 100 μM 8-Br-cGMP, or 2.5 μM LY83583 for various treatments for 3 days. Mean values and SE were calculated from three independent experiments (*n* = 20). Within each set of experiments, bars with different letters were signiﬁcantly different at the 0.05 level.

To evaluate the genetic mechanism of cGMP responses, we compared the primary root growth of WT (Col-0) seedlings and the *tir1-1*, *axr1-3*, and *axr1-12 Arabidopsis* mutants. TIR1 is an auxin receptor, and *Arabidopsis tir1-1* mutant shows reduced sensitivity to auxin (Ruegger *et al.*, 1998); *axr1* (*auxin-resistant 1*) mutants also exhibit a severe reduction in auxin response (Lincoln *et al.*, 1990). As shown in Fig. 5, exogenous 2,4-dichlorophenoxyacetic acid (2,4-D) markedly inhibited the primary root elongation (39.1% of the control) in WT (Col-0) seedlings. Furthermore, application of 8-Br-cGMP alleviated (increased by 31.7% of the 2,4-D treatment) the inhibition effect of 2,4-D in primary root elongation while LY83583 reinforced (reduced by 45.2% of the 2,4-D treatment) such inhibition (Fig. 5). Conversely, exogenous 8-Br-cGMP treatment did not relieve 2,4-D-inhibited primary root elongation in these mutants; moreover, these mutants also displayed reduced sensitivity to LY83583. Primary root elongation only decreased by 36.3, 10.7, and 18.1% under 2,4-D plus LY83583 treatment in *tir1-1*, *axr1-3*, and *axr1-12* mutants, respectively, compared with 2,4-D treatment alone (Fig. 5). TIR1 and AXR1 are the regulatory components of the SCF^TIR1/AFB^ complex (Schenck *et al.*, 2010), suggesting that the effect of cGMP on auxin-dependent primary root elongation might be involved in the process of SCF^TIR1/AFB^ signalling.

### cGMP enhances the expression of primary auxin-responsive genes

**Fig. 6.**
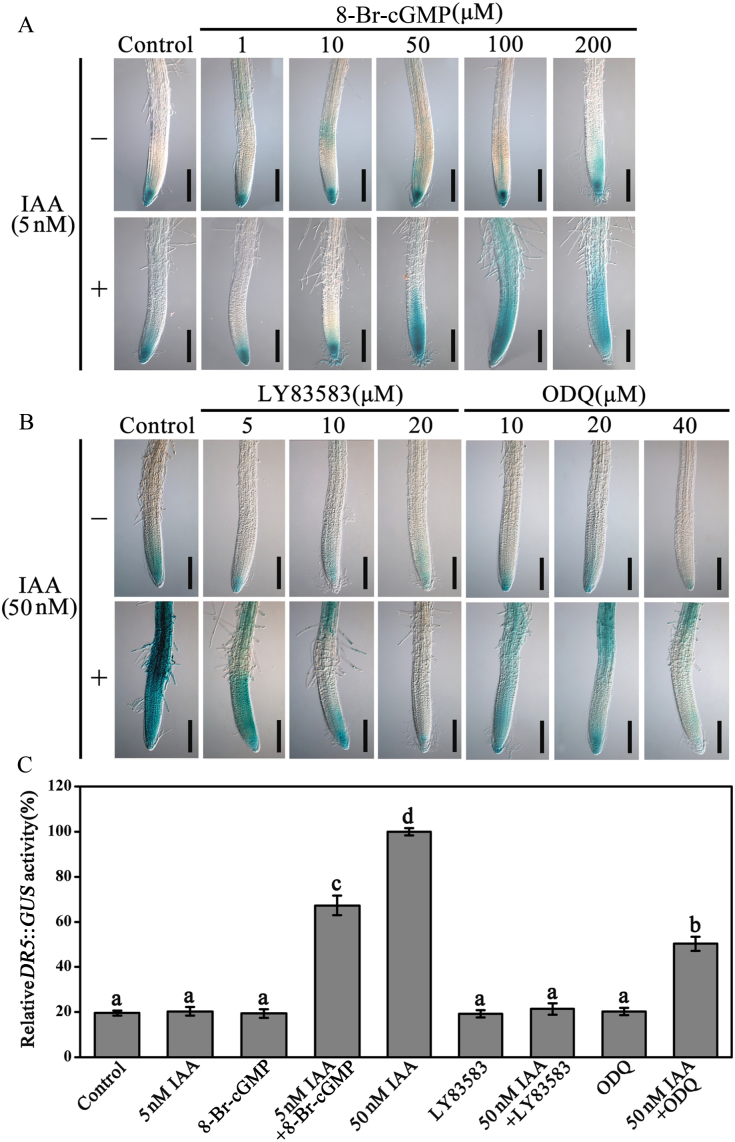
cGMP modulates auxin-induced *DR5*::*GUS* gene expression (A, B). Five-day-old seedlings were treated with 8-Br-cGMP with or without 5nM IAA for 6h (A) or treated with LY83583 or ODQ with or without 50nM IAA for 6h. For IAA plus LY83583 or ODQ treatments, the inhibitors were used to pretreat seedlings for 0.5h and then auxin was added for 6h (B). (C) Quantification of the GUS activity in *DR5*::*GUS* seedlings. The 7-day-old seedlings were treated with 5 or 50nM IAA and/or 100 μM 8-Br-cGMP, 20 μM LY83583, or 40 μM ODQ as in A and B. The GUS activity induced by 50nM IAA was adjusted to 100%. Mean values and SE were calculated from three independent experiments (*n* = 20). Within each set of experiments, bars with different letters were significantly different at the 0.05 level. Scale bars, 200 μm. This figure is available in colour at JXB online.

**Fig. 7.**
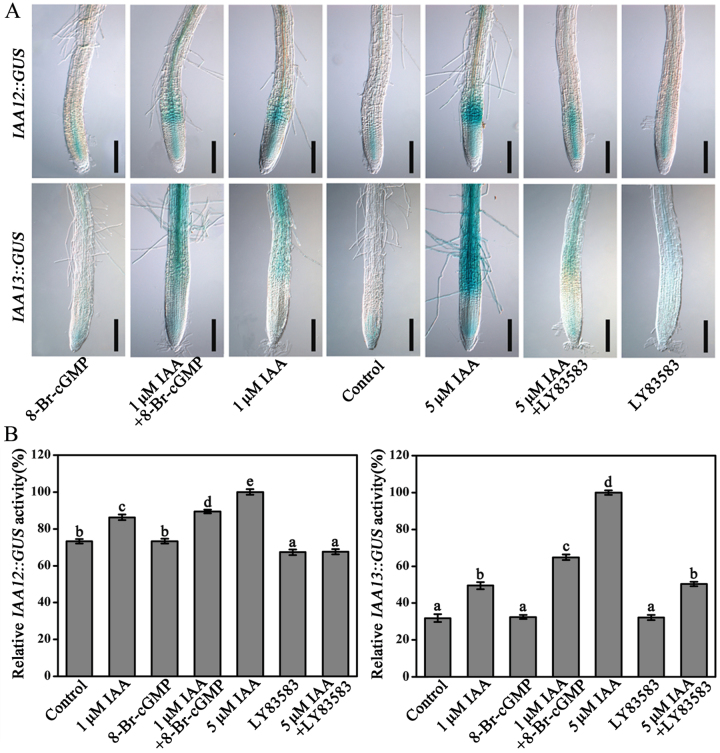
(A) Effects of cGMP on auxin-induced GUS activity in 5-day-old transgenic lines *IAA12*::*GUS* and *IAA13*::*GUS*. (B) Quantification of the GUS activity in roots of 7-day-old *IAA12*::*GUS* and *IAA13*::*GUS* seedlings. Seedlings were treated with 1 or 5 μM IAA, 100 μM 8-Br-cGMP, or 20 μM LY83583 as in Fig. 6 for 12h and the time of LY83583 pretreatment was 1h. The *IAA12*::*GUS* and *IAA13*::*GUS* activity induced by 5 μM IAA was adjusted to 100%. Mean values and SE were calculated from three independent experiments (*n* = 20). Within each set of experiments, bars with different letters were signiﬁcantly different at the 0.05 level. Scale bars, 200 μm. This figure is available in colour at JXB online.

To gain additional insights into the roles of cGMP in auxin signalling responses we employed the transgenic line *DR5*::*GUS*, which carries auxin-response elements fused to the β-glucuronidase-encoding gene (*GUS*). *GUS* was expressed poorly under a low concentration of IAA (5nM) treatment as well as with 8-Br-cGMP treatment alone (Fig. 6A). However, the simultaneous application of IAA and 8-Br-cGMP significantly promoted *GUS* gene expression, and 8-Br-cGMP increased the effect of IAA in a dosage-dependent manner (Fig. 6A). Both LY83583 and ODQ, a GC inhibitor, effectively inhibited the GUS activity induced by 50nM IAA (Fig. 6B). In addition, the transgenic lines *IAA12*::*GUS* and *IAA13*::*GUS* (Weijers *et al.*, 2005), in which *GUS* was driven by native auxin-inducible promoters, were also selected. As shown in Fig. 7, the GUS activity showed a similar profile as in Fig. 6 under different treatments in roots for *IAA12*::*GUS* and *IAA13*::*GUS* seedlings. Furthermore, the mode of GUS expression was further conﬁrmed by quantification (Figs 6C and 7B).

**Fig. 8.**
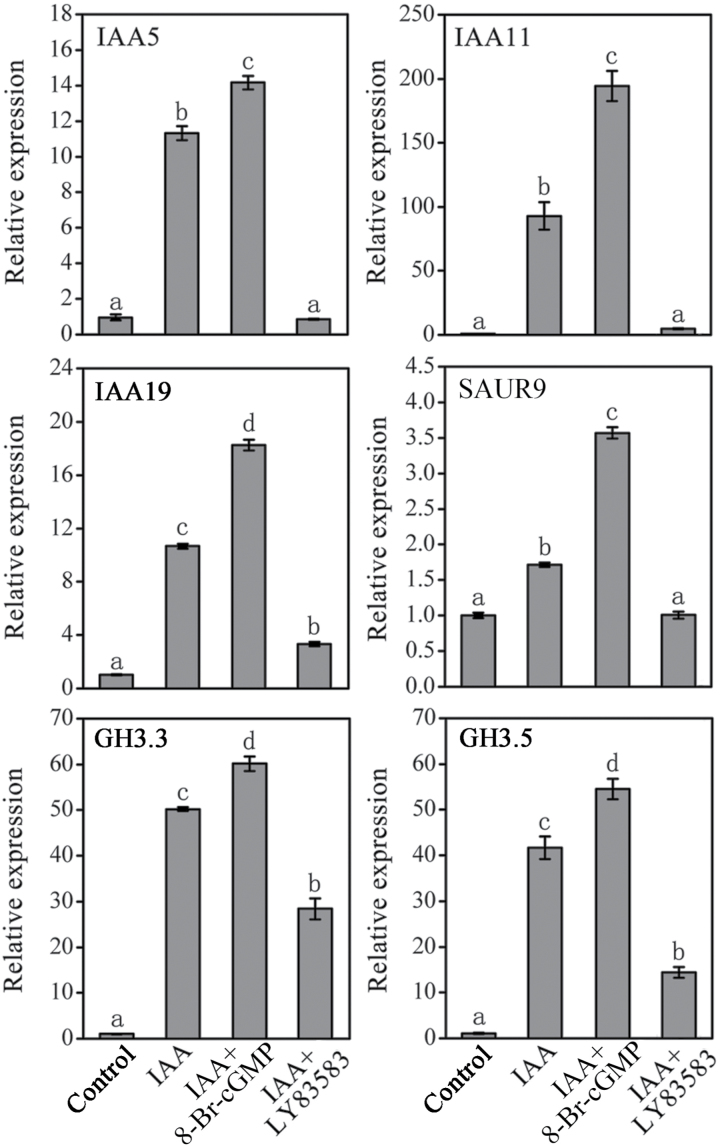
Quantitative real-time PCR analysis of the relative transcription abundance of auxin-responsive genes in WT seedlings. The 7-day-old seedlings were treated with 1 μM IAA with or without 100 μM 8-Br-cGMP or 20 μM LY83583 as in Fig. 6 for 6h. All gene transcripts were normalized to *ACTIN2*. Each of three independent cDNA preparations was assayed twice. Within each set of experiments, bars with different letters were signiﬁcantly different at the 0.05 level.

In order to clarify the role of cGMP in the expression of primary auxin-responsive genes, a set of auxin-response genes (*IAA5*, *IAA11*, *IAA19*, *SAUR9*, *GH3.3*, and *GH3.5*) was selected and analysed in *Arabidopsis* roots. With 1 μM IAA treatment for 6h the expression of these genes quickly increased (Fig. 8). The co-treatment of IAA and 8-Br-cGMP led to more marked increase of the expression of these genes in comparison with the IAA treatment alone (Fig. 8). However, the IAA-induced gene expression was effectively inhibited by the addition of LY83583 (Fig. 8). These results suggested that cGMP is indeed required for the expression of primary auxin-responsive genes.

### cGMP stimulates auxin-induced Aux/IAA degradation via the SCF^TIR1^ complex

**Fig. 9.**
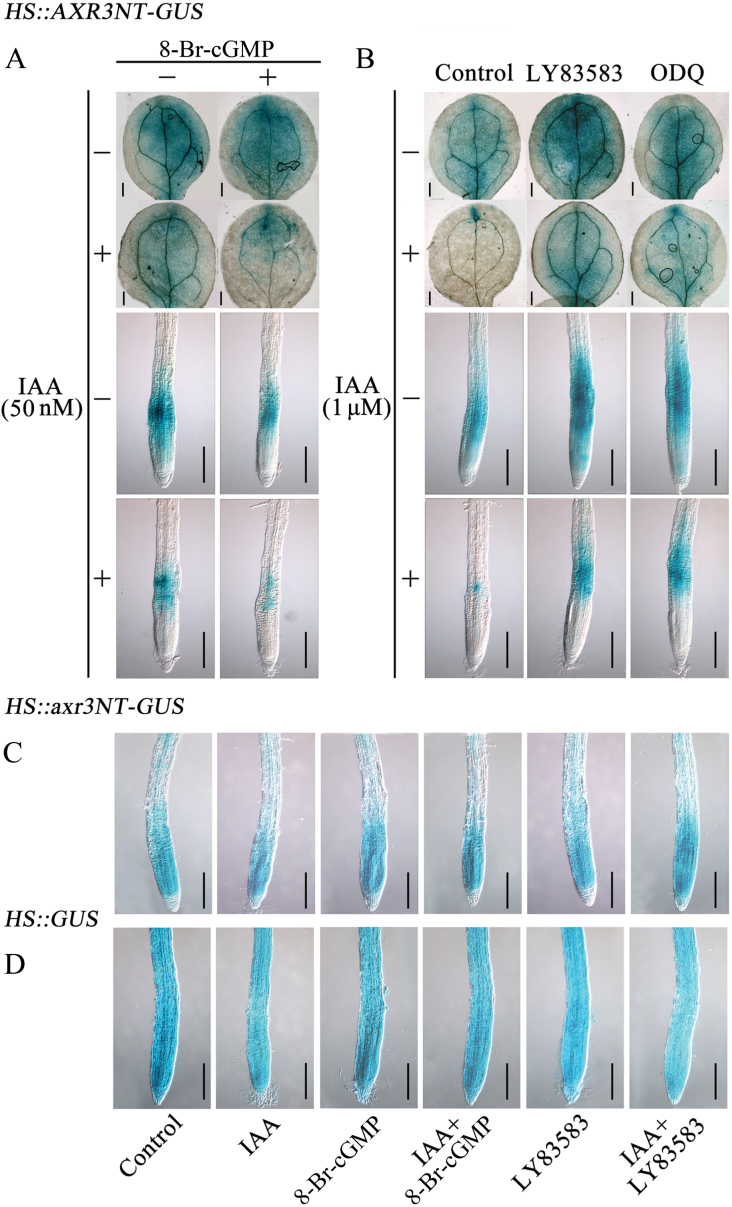
Effects of cGMP on auxin-dependent degradation of Aux/IAA protein. The 7-day-old seedlings of *HS::AXR3NT-GUS* (A, B), *HS::axr3-1NT-GUS* (C) and *HS::GUS* (D) were incubated in 1/2MS liquid medium at 37 °C for 2h, and then transferred into 1/2 MS liquid medium at 23 °C for 20min. After that, seedlings were treated with 50nM IAA, 1 μM IAA, 100 μM 8-Br-cGMP, 20 μM LY83583, and/or 40 µM ODQ in 1/2 MS liquid medium at 23 °C as in Fig. 6 for 1h. The time of LY83583 or ODQ pretreatment was 10min. Individual representative leaves and roots were photographed from three independent experiments with at least 30 seedlings examined for each experiment. Scale bars, 200 μm. This figure is available in colour at JXB online.

Auxin-mediated gene expression is regulated via degradation of Aux/IAA repressors (Gray *et al.*, 2001). Thus, the stability of the reporter protein AXR3NT-GUS was investigated using the *Arabidopsis* transgenic line *HS*::*AXR3NT*-*GUS* under various treatments. This reporter is a fusion of the N-terminus of the Aux/IAA protein AXR3/IAA17 (AXR3NT) and GUS under the control of a heat-shock inducible promoter (HS) (Gray *et al.*, 2001). As shown in Fig. 9A, 50 nM IAA caused a decrease in the AXR3NT-GUS stability in both leaves and roots of *HS*::*AXR3NT*-*GUS* plants, which was substantially enhanced by 8-Br-cGMP (Fig. 9A). Moreover, seedlings treated with LY83583 or ODQ and in combination with high IAA concentration (1 μM) exhibited much stronger GUS staining (Fig. 9B). To further confirm these results, two control lines, *HS*::*axr3-1NT-GUS*, in which the mutation in domain II of AXR3 results in an increased stability of the protein, and *HS*::*GUS* transgenic line were used. In contrast to *HS*::*AXR3NT-GUS* line, the GUS activity in *HS*::*axr3-1NT-GUS* and *HS*::*GUS* was unaffected by 8-Br-cGMP or LY83583 treatment in the presence or absence of auxin (Fig. 9C, D). These results suggested that cGMP might be involved in the auxin-mediated degradation of Aux/IAA proteins through SCF^TIR1^ proteasome pathway.

**Fig. 10.**
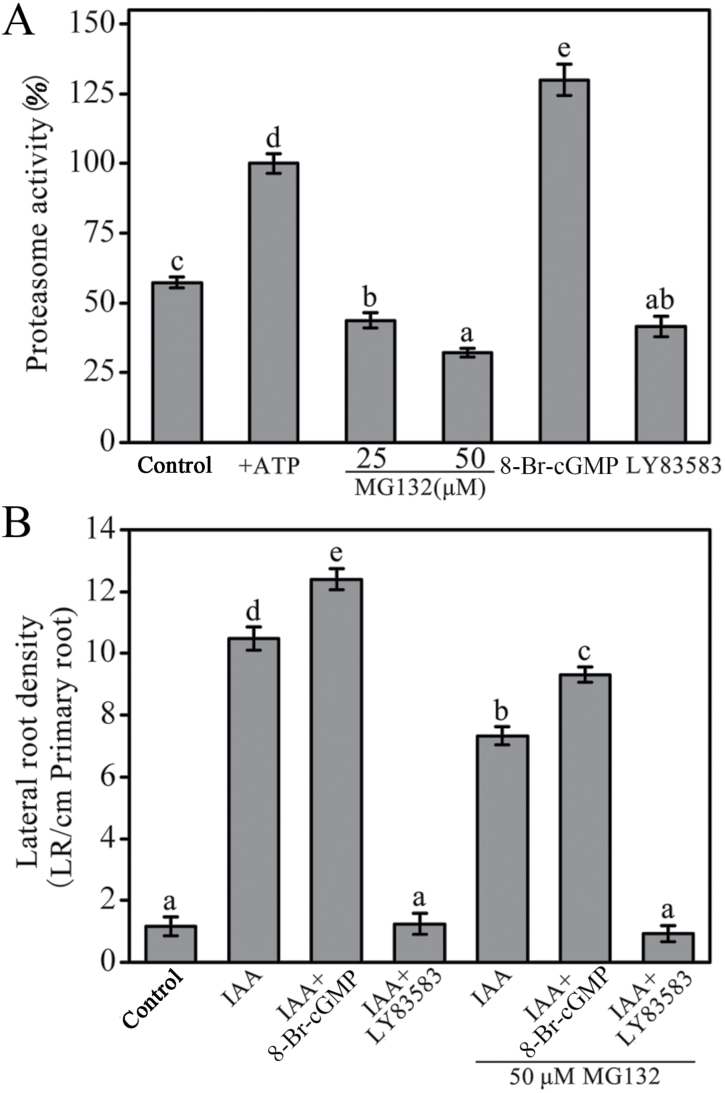
Roles of ATP-dependent 26S proteasome activity on cGMP-mediated auxin signalling. (A) cGMP changes the proteasome activity in roots of 7-day-old WT seedlings. Seedlings were treated with 100 μM 8-Br-cGMP, 20 μM LY83583, or MG132 for 12h. The proteasome activity enhanced by ATP in reaction solution was adjusted to 100%. (B) Auxin-induced LR formation was regulated by cGMP in a proteasome-independent way. Seedlings were grown on the medium containing 0.5 μM NPA for 5 days to repress LR initiation, and then they were treated with 1 μM IAA, 100 μM 8-Br-cGMP, 20 μM LY83583, and/or 50 μM MG132 for another 5 days. Mean values and SE were calculated from three independent experiments (*n* = 3). Within each set of experiments, bars with different letters were significantly different at the 0.05 level.

Pretreatment with 50 µM MG132 (the proteasome inhibitor) inhibits the degradation of the AXR3NT-GUS fusion protein and ubiquitin-ligase complex SCF^TIR1/AFB^-dependent responses (Dharmasiri *et al.*, 2005*b*; Robert *et al.*, 2010). Hence, we further investigated whether the roles of cGMP on auxin-induced degradation of Aux/IAA proteins were related to the 26S proteasome and then the ATP-dependent proteasome activity was determined using a ﬂuorogenic peptide substrate (Fujinami *et al.*, 1994) in *Arabidopsis* roots. As shown in Fig. 10, proteasome activity was significantly promoted (increased to 129.9%) by 8-Br-cGMP but seriously inhibited (decreased to 41.6%) by LY83583 in the presence of ATP (Fig. 10A). As a positive control, 50 µM MG132 completely repressed the activity of the 26S proteasome (Fig. 10A). These results indicated that cGMP on auxin-induced degradation of Aux/IAA proteins might be achieved by regulating 26S proteasome activity. However, subsequent results showed that application of MG132 reduced auxin-induced LR formation, but it did not alter the effect of 8-Br-cGMP or LY83583 (Fig. 10B). Therefore, we propose that cGMP promotes auxin-induced LR formation by a proteasome-independent mechanism.

### The effect of cGMP on the auxin-enhanced TIR1–Aux/IAA interaction

**Fig. 11.**
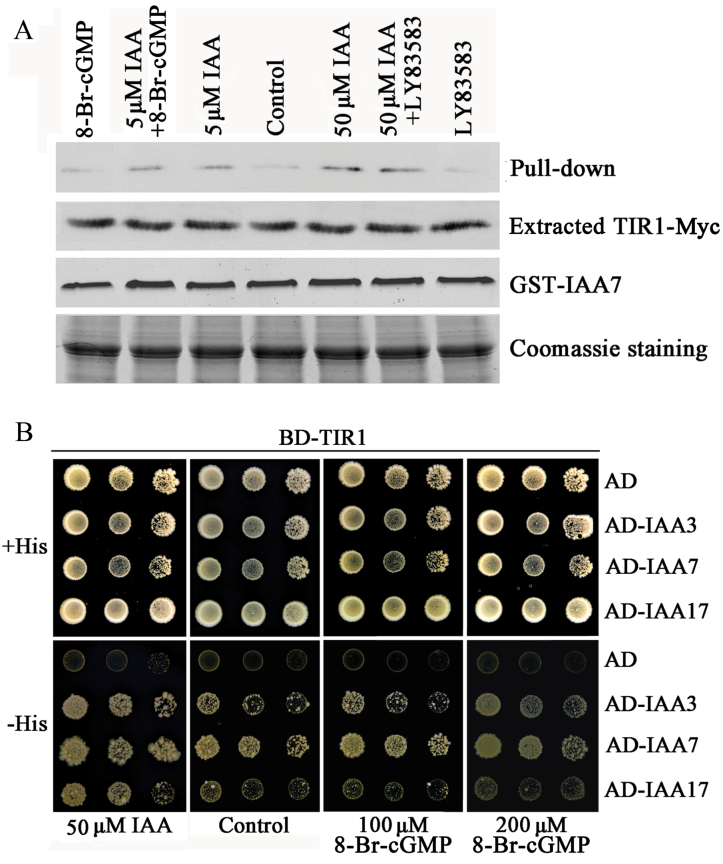
cGMP does not affect the auxin-enhanced TIR1–Aux/IAA interaction. (A) Pull-down reactions were performed through anti-c-Myc immunoblot analysis. Reactions were carried out using protein extracts from 7-day-old *tir1-1*[TIR1-Myc] seedlings expressing TIR1-Myc and from bacterium producing GST-IAA7/AXR2 in the presence of the indicated IAA concentration and 100 μM 8-Br-cGMP or 20 μM LY83583. The extracted TIR1-Myc and GST-IAA7 gels show that equal amounts of TIR1 and IAA7 proteins were put into the pull-down reactions. Coomassie blue-stained gel was used as loading control. The experiments were repeated at least three times. (B) Yeast two-hybrid assay of the interaction between TIR1 and Aux/IAA proteins. Yeast two-hybrid assays were carried out with cells co-transformed with the indicated plasmids and grown on SD/−Trp/−Leu/−His selective media plus the addition of 50 µM IAA or two different concentrations of 8-Br-cGMP. This figure is available in colour at JXB online.

Auxin treatment stimulates the interaction between SCF^TIR1^ and Aux/IAA proteins and promotes their degradation via the 26S proteasome, therefore inducing the expression of primary auxin-responsive genes (Gray *et al.*, 2001; Kepinski and Leyser, 2005). To analyse whether cGMP affects the auxin-dependent TIR1–Aux/IAA interaction, we performed GST pull-down experiments using IAA7/AXR2 and Myc-tagged TIR1 proteins. However, results showed that 8-Br-cGMP was not able to stimulate the interaction of TIR1-Myc and GST-IAA7/AXR2. Furthermore, LY83583 also did not block the IAA-enhanced interaction between TIR1-Myc and GST-IAA7/AXR2 (Fig. 11A). Next, to further verify the above conclusion, we investigated the effect of cGMP on *in vivo* TIR1–Aux/IAA interactions using the *GAL4* two-hybrid system (Bian *et al.*, 2012). In yeast cells 8-Br-cGMP was also unable to influence the interaction of TIR1 with IAA/AUXs (IAA3, IAA7, and IAA17) compared with IAA (Fig. 11B). Altogether, *in vitro* and *in vivo* experiments indicated that cGMP is unable to directly influence TIR1–Aux/IAA interactions.

### cGMP-mediated modulation of auxin signalling through PKG activity

**Fig. 12.**
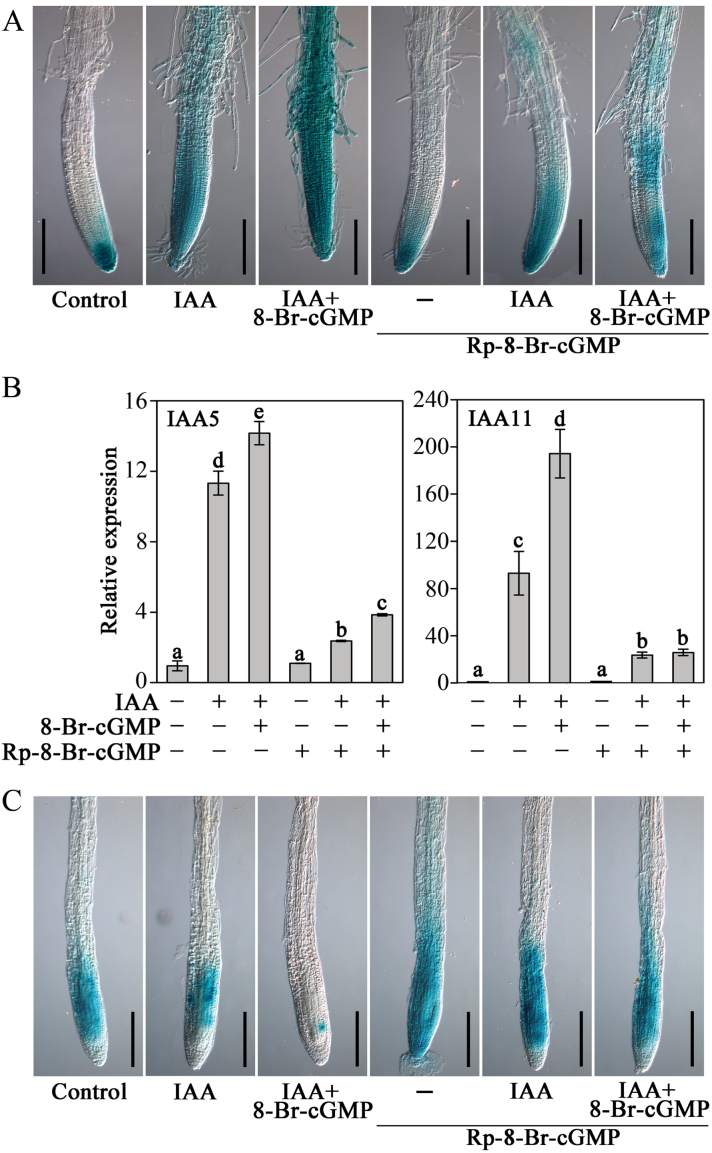
Effects of PKG inhibitor Rp-8-Br-cGMP on the expression of auxin-responsive genes and auxin-dependent degradation of Aux/IAA protein. (A) GUS activity analysis in 5-day-old *DR5*::*GUS* seedlings. (B) Quantitative real-time PCR analysis of the expression of auxin-responsive genes (*IAA5*, *IAA11*) in 7-day-old WT seedlings, normalized to *ACTIN2*. (C) GUS activity analysis in 7-day-old *HS*::*AXR3NT*-*GUS* seedlings. In A and B seedlings were pretreated with 100 μM Rp-8-Br-cGMP for 0.5h before adding 50nM IAA or 100 μM 8-Br-cGMP treatment for 6h. In C seedlings were incubated in 1/2 MS liquid medium at 37 °C for 2h and then transferred into 1/2 MS liquid medium at 23 °C for 20min. After that, the seedlings were pretreated with 100 μM Rp-8-Br-cGMP for 10min before adding 1 μM IAA or 100 μM 8-Br-cGMP for 1h in 1/2 MS liquid medium at 23 °C. Individual representative roots were photographed from three independent experiments with at least 30 seedlings examined for each experiment, and bars with different letters were signiﬁcantly different at the 0.05 level. Scale bars, 200 μm.This figure is available in colour at JXB online.

As reported previously, PKG, consisting of a cyclic nucleotide-binding domain and a protein kinase domain, is a typical downstream cellular target or effector of cGMP modulation in animals and plants (Newton and Smith, 2004; Maathuis, 2006). Therefore, cGMP may affect auxin signalling via PKG action. To address this possibility we used Rp-8-Br-cGMP, a putative inhibitor of PKG. This reagent selectively inhibits the PKG activity in animal cells and auxin-induced stomatal opening in *Arabidopsis* (Butt *et al.*, 1990; Cousson, 2010). As shown in Fig. 12A and B, we observed that pretreatment with 100 μM Rp-8-Br-cGMP blocked expression – induced by either IAA alone or IAA plus 8-Br-cGMP – of these auxin-responsive genes, including *DR5*::*GUS* reporter, *IAA5*, and *IAA11*. In addition, it also seriously abolished the degradation – induced by IAA alone or IAA plus 8-Br-cGMP – of the AXR3NT-GUS fusion protein (Fig. 12C). These results strongly suggest that cGMP-mediated modulation of auxin signalling is dependent on the PKG activity in *Arabidopsis*.

## Discussion

Previous studies have demonstrated that the plant growth regulator auxin and the second messenger cGMP coordinate several developmental and physiological processes, including adventitious root development and stomatal opening (Meier and Gehring, 2006). In this study we have provided evidence that cGMP, as a positive modulator, is involved in the auxin-regulated signalling response in *Arabidopsis* roots, which depends on PKG activity.

As previously reported in other plant species, our results showed that IAA induces endogenous cGMP accumulation in *Arabidopsis* roots (Fig. 1A). A recent study using the ﬂuorescent reporter FlincG as an endogenous cGMP sensor also showed that auxin rapidly increased cellular cGMP within a short period of time in *Arabidopsis* protoplast (Isner *et al.*, 2012), and which was consistent with our results. Moreover, IAA could markedly induce GC activity in a concentration-dependent manner in *Arabidopsis* roots (Fig. 1B). It has been reported that cGMP accumulation was attributable to activation of GC rather than 3′,5′-cyclic-cGMP phosphodiesterase in ABA-mediated stomatal movement (Dubovskaya *et al.*, 2011). These results suggest that auxin could increase the endogenous cGMP levels by affecting GC activity in *Arabidopsis* roots.

Auxin is a key phytohormone involved in a broad spectrum of developmental and physiological processes in plants, where it notably contributes to the regulation of root-system architecture remodelling (Woodward and Bartel, 2005; Vanneste and Friml, 2009). In this study, the LR density induced by auxin was obviously strengthened by 8-Br-cGMP and completely suppressed by LY83583, suggesting that cGMP is required for auxin-stimulated LR formation (Fig. 2). This was further conﬁrmed by the assay of stained LR sites using GUS in the transgenic line *DR5::GUS* (Fig. 2B). These results agreed with the previous conclusion that cGMP is involved in auxin-mediated adventitious root formation (Pagnussat *et al.*, 2003; Bai *et al.*, 2012). Furthermore, we presented evidence that cGMP is also involved in auxin-regulated root hair development (Fig. 3) and primary root growth (Figs 4 and 5). The root-elongation assay showed that cGMP could rescue the inhibition of primary root growth by exogenous auxin while LY83583 exacerbated it. However, the phenomenon was not obviously observed in the *tir1-1*, *axr1-3*, and *axr1-12* mutants (Figs 4 and 5). Moreover, *axr1-3* and *axr1-12* mutants exhibited fewer LRs and reduced root hair formation (del Pozo *et al.*, 2002; Swarup *et al.*, 2002), suggesting that cGMP might modulate auxin-induced root growth by affecting the auxin signal response.

Auxin regulates plant development by inducing rapid cellular responses and changes in gene expression (Gray *et al.*, 2001). Here, we found that 8-Br-cGMP enhanced auxin-induced expression of the auxin reporter gene *DR5*::*GUS*; this was effectively inhibited by treatment with the GC inhibitors ODQ or LY83583 (Fig. 6). Furthermore, this phenomenon were also supported by the native auxin-inducible promoters *IAA12*::*GUS* and *IAA13*::*GUS* (Fig. 7) and the mRNA levels of primary auxin-induced genes (*Aux/IAA*s, *GH3*s, and *SAUR*s) in *Arabidopsis* roots (Fig. 8). Therefore, in agreement with the physiological data, these results clearly indicated that cGMP is also required for expression of auxin-responsive genes. It has been reported that *Arabidopsis* F-box proteins TIR1/AFB are auxin receptors that mediate degradation of Aux/IAA repressors to induce auxin-regulated responses (Gray *et al.*, 2001; Dharmasiri *et al.*, 2005*a*; Kepinski and Leyser, 2005). Auxin alters the stability of Aux/IAA repressors, and therefore cGMP may act by affecting Aux/IAA protein degradation, thus explaining the effects of cGMP on the expression of primary auxin-responsive genes. To test this possibility we used the *HS*::*AXR3NT-GUS* transgenic line, which strongly expresses an AXR3/IAA17 translational fusion protein under the control of a heat-shock promoter, to analyse the effect of cGMP on auxin-induced degradation of Aux/IAA proteins (Gray *et al.*, 2001). We found that 8-Br-cGMP accelerated the degradation rate of the Aux/IAA fusion protein caused by IAA treatment (Fig. 9A). In contrast, treatment with LY83583 or ODQ abolished degradation of the fusion protein in the presence or absence of IAA (Fig. 9B). These results suggested that cGMP might regulate the expression of primary auxin-responsive genes by activating auxin-induced Aux/IAA degradation.

It is well known that MG132 blocks the degradation of Aux/IAA protein by repressing the proteasome activity in *Arabidopsis* (Gray *et al.*, 2001). Similarly to MG132, our results showed that the inhibition of cGMP synthesis strongly inhibited the degradation of Aux/IAA protein (Fig. 9). Therefore, we further examined whether the effects of cGMP was related to the ATP-dependent 26S proteasome activity. It was shown that exogenous 8-Br-cGMP increased and LY83583 strongly suppressed the ATP-dependent proteasome activity in *Arabidopsis* roots, respectively (Fig. 10A). These results further confirmed that cGMP acted on the auxin signalling pathway through the SCF^TIR1^-mediated degradation of Aux/IAAs. However, we found that repression of the proteasome activity using MG132 could not block the action of cGMP on IAA-induced LR formation in *Arabidopsis* roots, suggesting that cGMP promotes auxin-induced LR formation by a proteasome-independent mechanism (Fig. 10B). These seemingly paradoxical results of cGMP action are similar to nitric oxide (NO), which was reported to be involved in the auxin signalling through Aux/IAA degradation (Terrile *et al.*, 2012), whereas it promotes reduction of PIN1 protein levels by a proteasome-independent mechanism (Fernández-Marcos *et al.*, 2011). In addition, we also noticed that the inhibition of proteasome activity could not entirely repress the effect of auxin on LR formation (Fig. 10B), suggesting that the effect of cGMP on auxin-induced LR formation might be involved much more complicated mechanisms. Taking into account all these ﬁndings, we propose that cGMP might operate in multiple ways, including the dependent as well as independent regulation of proteasome-dependent Aux/IAA ubiquitination and subsequent degradation.

TIR1 as the auxin-recognition component of the SCF complex that interacts with Aux/IAA proteins to target them for proteolysis has been illustrated in detail (Gray *et al.*, 2001; Dharmasiri *et al.*, 2005*a*; Kepinski and Leyser, 2005). Recent work has shown that the amount of endogenous TIR1 protein appeared to be rate-limiting for auxin response and excess TIR1 protein in *35S::TIR1* even led to the degradation of Aux/IAAs in the absence of auxin treatment (Maraschin *et al.*, 2009). In addition, the discovery that inositol hexakisphosphate is associated with the TIR1 protein (Tan *et al.*, 2007) suggests that TIR1 activity might be regulated by additional cofactors. All these findings suggest that TIR1 activity and its interaction with Aux/IAA proteins play a crucial role in auxin responses. Hence we focused on the effect of cGMP on interaction between TIR1 and Aux/IAA proteins. However, further molecular evidence showed that cGMP could not alter the auxin-enhanced interaction between TIR1 and Aux/IAAs, suggesting that cGMP did not directly alter the binding between TIR1 and its ligand, auxin (Fig. 11). Previous studies have shown that TIR1 is a member of a small gene family that contains ﬁve additional AFB proteins that all function as auxin receptors (Dharmasiri *et al.*, 2005*b*; Greenham *et al.*, 2011). The functional defects of TIR1 protein evoke the reduction in auxin response. Moreover, the SCF complex mutants *axr1-3* and *axr1-12* also showed alteration of responses to auxin (del Pozo *et al.*, 2002; Swarup *et al.*, 2002) and Aux/IAA proteins exhibit increased stability in *axr1* and *tir1* mutants (Gray *et al.*, 2001). In addition, our results showed that the *tir1-1*, *axr1-3*, and *axr1-12* mutants displayed reduced sensitivity to LY83583 and 8-Br-cGMP on auxin-inhibited primary root elongation (Fig. 5), suggesting that cGMP might be involved in SCF^TIR1/AFB^ signalling. Thus, it is possible that cGMP alters the interaction of other AFB proteins with Aux/IAA proteins or TIR1–Aux/IAA interaction via downstream effectors of cGMP signalling, such as PKG.

It is well known that PKG is also a regulator of protein activation. Thus, it is plausible that cGMP inﬂuences TIR1–Aux/IAA interaction through the PKG and that PKG is able to modify TIR1 activity, although findings from this study cannot directly prove this issue. However, it is worth addressing in a future work. In order to further test the hypothesis, the putative inhibitor of PKG, Rp-8-Br-cGMP, was used to examine whether the cGMP-mediated expression of auxin-responsive genes and degradation of the AXR3NT-GUS fusion protein is dependent on PKG activity. As expected, our data showed that the inhibition of PKG activity strongly blocked the auxin responses (Fig. 12), suggesting that cGMP might inﬂuence auxin signalling in *Arabidopsis* roots through PKG activity. This conclusion is in agreement with previous findings that cGMP as a mediator participates in photoperiodic ﬂowering induction in *Pharbitis nil* (Szmidt-Jaworska *et al.*, 2009) and auxin-induced stomatal opening in *Arabidopsis* via its inﬂuence on PKG activity (Cousson, 2010). In addition, it has been demonstrated that AGC kinases are required for auxin-related processes such as auxin-mediated root development and organogenesis in *Arabidopsis* (Galván-Ampudia and Offringa, 2007; Cheng *et al.*, 2008). PKG is a component of AGC kinases, which further complement our conclusion that the cGMP-mediated auxin response is dependent on PKG activity.

**Fig. 13.**
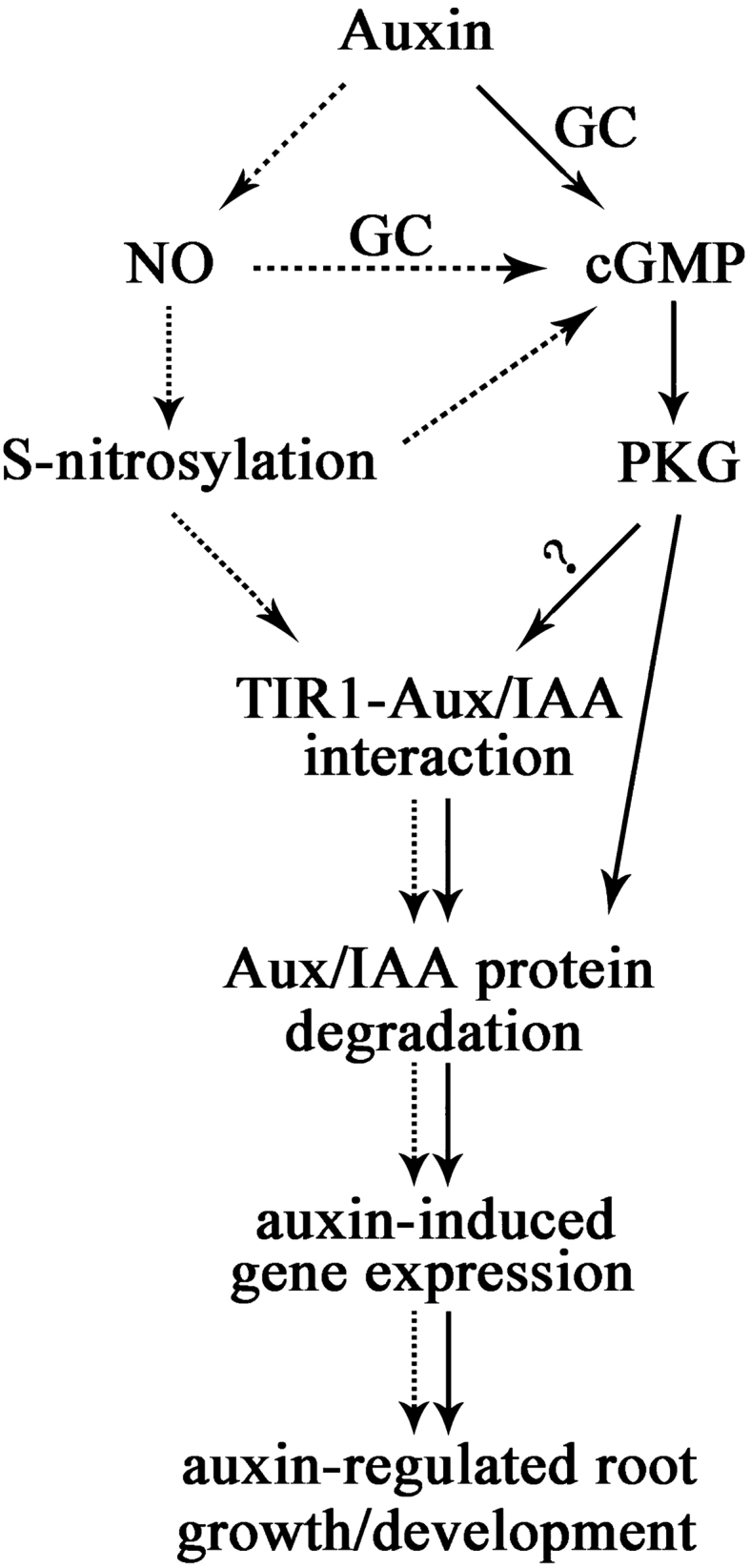
Schematic illustration of a proposed model for the link between NO, cGMP, and auxin signalling in *Arabidopsis* roots. In this model, solid arrows indicate results from this paper and dotted arrows indicate results from the literature.

In summary, our results have revealed a new aspect of cGMP signalling, and explained the mechanism of the involvement of cGMP in the auxin signalling pathway in *Arabidopsis* root development. We propose one possible model in Fig. 13. According to this model, although the direct cellular targets of cGMP action remain unknown, several lines of evidence indicate that treatment with auxin in the roots can rapidly induce the accumulation of cGMP by stimulating GC activity; cGMP then influences the auxin-dependent SCF^TIR1^ complex through PKG action, which results in Aux/IAA degradation, facilitates activation of gene expression, and finally affects auxin-regulated root growth. It is well known that cGMP is an important component of NO signalling and a number of NO-regulated physiological processes may be mediated by GC (Wu *et al.*, 2013). Recently, similar to cGMP, Terrile *et al.* (2012) reported that NO and its S-nitrosylation are involved in auxin signalling and regulate auxin-dependent gene expression, Aux/IAA protein degradation, and TIR1–Aux/IAA interaction (Fig. 13). However, whether they share the signalling pathway or have more complicated regulation mechanisms in auxin signalling needs future investigation.

## Supplementary material

Supplementary material is available at *JXB* online.

Table S1. Sequences of primers used in the study.

Table S2. The OD values of the control group in cGMP detection.

Table S3. The values of the standard curve in cGMP detection.

Figure S1. The standard curve used for cGMP detection.

Figure S2. Change of endogenous cGMP levels after various treatments in roots of 7-day-old WT seedlings: 100 μM 8-Br-cGMP, 20 μM LY83583, and 5 μM IAA were used for various treatments for 1h. For IAA plus LY83583 treatment, seedlings were pretreated with LY83583 for 10min and then treated with IAA plus LY83583 for 1h. Mean values and SE were calculated from three independent experiments.

Supplementary Data

## Abbreviations:

Aux/IAA: auxin/indole-3-acetic acid
8-Br-cGMP: 8-bromoguanosine 3′,5′-cyclic guanosine monophosphate
cGMP: cyclic guanosine 3′,5′-monophosphate
2,4-D: 2,4-dichlorophenoxyacetic acid
GC: guanylate cyclase
GST: glutathione-S-transferase
GUS: β-glucuronidase
IAA: indole-3-acetic acid
LR: lateral root
LY83583: 6-anilino-5,8-quinolinedione
1/2MS: half-strength Murashige and Skoog
NAA: 1-naphthaleneacetic acid
NPA: 1-naphthylphthalamic acid
ODQ: 1H-[1,2,4]-oxadiazole-[4,3-a]-quinoxalin-1-one
PKG: cGMP-dependent protein kinase
X-Gluc: 5-bromo-4-chloro-3-indolyl-β-d-glucuronic acid
WT: wild type.
