# Supplementary material for: Cyclic GMP is involved in auxin signalling during Arabidopsis root growth and development
Source: J Exp Bot. 2014 Mar 3;65(6):1571–83. doi: 10.1093/jxb/eru019 (PMC3967089; doi:10.1093/jxb/eru019)
Supplement: Supplementary Data [file supp_65_6_1571__index.html]

Cyclic GMP is involved in auxin signalling during Arabidopsis root growth and development — Cyclic GMP is involved in auxin signalling during Arabidopsis root growth and development — Supplementary Data 

# Cyclic GMP is involved in auxin signalling during *Arabidopsis* root growth and development

## Supplementary Data

Data files

**Files in this Data Supplement:**

- Supplementary Data - Supplementary Data
