# Supplementary material for: Cyclic GMP is involved in auxin signalling during Arabidopsis root growth and development
Source: J Exp Bot. 2014 Mar 3;65(6):1571–83. doi: 10.1093/jxb/eru019 (PMC3967089; doi:10.1093/jxb/eru019)
Supplement: Supplementary Data [file supp_eru019_jexbot109124_file001.pdf]

Table S1. Sequences of primers used in the study

Gene name	Accession number	fragment length(bp)	Primer sequence 5'-3'
Primers used for qRT-PCR			
AtACTIN2	*Atu41998*	137	TTTCCCGCTCTGCTGTTGT<br>TGTGCCAATCTACGAGGGTTT
AtIAA5	*At1g15580*	61	GCTCTGCAAATTCTGTTCGGA<br>ATTCACTTTCCTTCAACGTATCATCAA
AtIAA11	*At4g28640*	227	GGTCTTACGTTGAGCCTTGG<br>GTGGCTGAAGCCTTAGCTTG
AtIAA19	*At3g15540*	462	ATGGAGAAGGAAGGACTCGGGCTTG<br>GTCTTCGTATATGGTAACGTATTCGC
AtSAUR9	*At4g36110*	150	GACGTGCCAAAAGGTCACTT<br>AGTGAGACCCATCTCGTGCT
AtGH3.3	*At2g23170*	462	TCCACTAAGGACGTGAAGGCTCTAAG<br>TGCTGGTAATCCACCGGGAGTCTTCG
AtGH3.5	*At4g27260*	514	AGCCCTAACGAGACCATCCT<br>AAGCCATGGATGGTATGAGC
Primers used for Yeast two-hybrid			
AtTIR1	*At3g62980*	1795	GAATTCATGCAGAAGCGAATAGCCTTGTC<br>GGATCCATAATCCGTTAGTAGTAATGATTTGCCTG
AtIAA3	*At1g04240*	582	GAATTCATGGATGAGTTTGTTAACCTCAAGG<br>GGATCCTCATACACCACAGCCTAAACCTTTG
AtIAA7	*At3g23050*	640	GAATTCATCGGCCAACTTATGAACCTCAAGG<br>GGATCCACTCCCATGGGACATCGCCAAC
AtIAA17	*At1g04250*	699	GAATTCATGATGGGCAGTGTCGAGCTGAATC<br>GGATCCAGCTCTGCTCTTGCACTTCTCCATC

Table S2. The OD values of control group in cGMP detection

Control groups	Blank (zero control)	NSB	TA	B0
OD value	0	0.292776	0.000818	0.309276
OD value	0.001808	0.261166	0.000226	0.308328

Table S3. The values of standard curve in cGMP detection

Formula: **X axis:** Log10(C) **Y axis:** Ln(P/(1-P)) P= average OD/B0

cGMP Standards (C=pmol/ml)	100	20	4	0.8	0.16
OD value	0.005864	0.020971	0.069216	0.174969	0.262099
OD value	0.005223	0.021092	0.076089	0.180739	0.258461

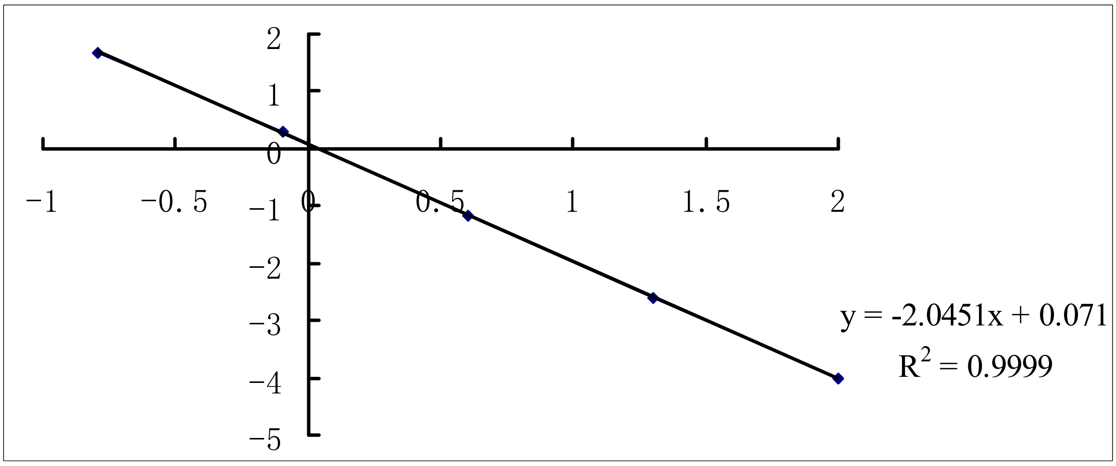

Figure S1. The standard curve used for cGMP detection

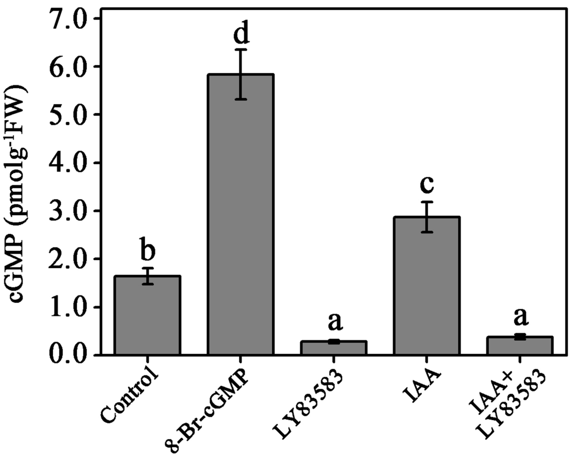

Figure S2. Change of endogenous cGMP levels after various treatments in roots of 7-day-old WT seedlings. 100 μM 8-Br-cGMP, 20 μM LY83583 and 5 μM IAA were used for various treatments for 1 h. For IAA plus LY83583 treatment, Seedlings were pretreated with LY83583 for 10 min, and then treated with IAA plus LY83583 for 1 h. Mean values and SE were calculated from three independent experiments.
